# Comparison of PET metabolic indices for the early assessment of tumour response in metastatic colorectal cancer patients treated by polychemotherapy

**DOI:** 10.1007/s00259-012-2274-x

**Published:** 2012-11-14

**Authors:** Jacques-Antoine Maisonobe, Camilo A. Garcia, Hatem Necib, Bruno Vanderlinden, Alain Hendlisz, Patrick Flamen, Irène Buvat

**Affiliations:** 1 IMNC UMR 8165 CNRS – Paris 7 and Paris 11 Universities, Building 440, Orsay Campus, 91406 Orsay Cedex, France; 2 Department of Nuclear Medicine, Institut Jules Bordet, Université Libre de Bruxelles, Brussels, Belgium; 3 Department of Gastroenterology, Institut Jules Bordet, Université Libre de Bruxelles, Brussels, Belgium

**Keywords:** Partial volume effect, Treatment response, SUV, Classification performances, FDG PET, Colorectal cancer

## Abstract

**Purpose:**

To compare the performance of eight metabolic indices for the early assessment of tumour response in patients with metastatic colorectal cancer (mCRC) treated with chemotherapy.

**Methods:**

Forty patients with advanced mCRC underwent two FDG PET/CT scans, at baseline and on day 14 after chemotherapy initiation. For each lesion, eight metabolic indices were calculated: four standardized uptake values (SUV) without correction for the partial volume effect (PVE), two SUV with correction for PVE, a metabolic volume (MV) and a total lesion glycolysis (TLG). The relative change in each index between the two scans was calculated for each lesion. Lesions were also classified as responding and nonresponding lesions using the Response Evaluation Criteria In Solid Tumours (RECIST) 1.0 measured by contrast-enhanced CT at baseline and 6–8 weeks after starting therapy. Bland-Altman analyses were performed to compare the various indices. Based on the RECIST classification, ROC analyses were used to determine how accurately the indices predicted lesion response to therapy later seen with RECIST.

**Results:**

RECIST showed 27 responding and 74 nonresponding lesions. Bland-Altman analyses showed that the four SUV indices uncorrected for PVE could not be used interchangeably, nor could the two SUV corrected for PVE. The areas under the ROC curves (AUC) were not significantly different between the SUV indices not corrected for PVE. The mean SUV change in a lesion better predicted lesion response without than with PVE correction. The AUC was significantly higher for SUV uncorrected for PVE than for the MV, but change in MV provided some information regarding the lesion response to therapy (AUC >0.5).

**Conclusion:**

In these mCRC patients, all SUV uncorrected for PVE accurately predicted the tumour response on day 14 after starting therapy as assessed 4 to 6 weeks later (i.e. 6 to 8 weeks after therapy initiation) using the RECIST criteria. Neither correcting SUV for PVE nor measuring TLG improved the assessment of tumour response compared to SUV uncorrected for PVE. The change in MV was the least accurate index for predicting tumour response.

## Introduction

PET/CT is a promising tool for detecting molecular signals associated with tumour response soon after therapy initiation (e.g. [1–4]). To help standardize procedures and achieve comparable quantitative measurements among institutions using ^18^F-FDG PET/CT, guidelines and recommendations are being been proposed [5–7]. Yet, there is still a lack of consensus as to which index to use to characterize tumour metabolism. The European Organization for Research and Treatment of Cancer recommends the use of the metabolic glucose rate derived from a kinetic analysis and based on the measurement of the time-course of radioactivity in tissue and arterial blood, or the mean or maximum standardized uptake value (SUV) normalized to body surface area [5]. PERCIST 1.0 [6] advocates the use of an SUV normalized to lean body mass (SUL) computed in a small sphere (about 1 cm^3^) including the tumour voxel of maximum intensity so that the mean value in the sphere is maximized (SUL_peak_). PERCIST 1.0 also suggests reporting the maximum SUL in the tumour, the mean SUL in volumes containing voxels with SUL greater than 50 % or 70 % of SUL_peak_ and/or the total lesion glycolysis (TLG [8]). Although the maximum SUV in the tumour (SUV_max_) is by far the most reported index [6, 9], the most relevant index in the context of patient monitoring remains to be identified. It has been shown that the accuracy, robustness, classification performance and test–retest variability of semiquantitative indices greatly depend on the definition of the index [10–13]. Cheebsumon et al. [14] recently showed that absolute quantitation using metabolic glucose rate might yield an interpretation different from that based on SUV in the context of patient monitoring. The role of the TLG index, which includes information regarding the metabolically active volume (MV) and the uptake in this volume, also needs to be clarified [15, 16].

The partial volume effect (PVE) is one of the main sources of error in the quantitative characterization of tumour metabolism in FDG PET/CT [16]. The way it is dealt with might have an impact on early tumour response assessment [17, 18]. Indeed, due to PVE, SUV often reflects both the metabolic activity and the MV [19], especially in small lesions. The severity of PVE can be reduced by modelling the imaging system point spread function during the reconstruction process [20, 21]. PVE can also be compensated for by postprocessing the reconstructed images [22] or the values derived from those images [23, 24]. Recent reviews regarding the various approaches that might be used to correct for PVE are available [17, 19].

The aim of this study was to clarify the impact of PVE and PVE correction on the early assessment of tumour response in patients with metastatic colorectal cancer (mCRC) treated with polychemotherapy. We compared the performance of eight indices (four SUV indices without PVE correction, two SUVs compensated for PVE, a MV index and a TLG index) derived from a PET/CT scan performed 2 weeks after treatment initiation to predict the tumour response determined using the RECIST 1.0 criteria 6 to 8 weeks after treatment.

## Materials and methods

### Patients

Forty patients with advanced mCRC treated at the Institute Jules Bordet, Brussels, Belgium, were enrolled in the study. The patients’ characteristics are shown in Table 1. The patients were recruited as part of a prospective clinical trial in a larger cohort of patients, the aim of the clinical trial being to assess the clinical role of early FDG PET/CT scanning in chemotherapy-treated mCRC [13, 25]. The study was approved by the ethics committee of the Institute Jules Bordet and registered at clinicaltrials.gov (number NCT00741481). The patients’ treatment regimens are listed in Table 1. No targeted drugs (anti-VEGF, anti-EGFR) were used.

**Table 1:** Patient characteristics

Characteristic	Value
Total number of patients	40
Total number of lesions	101
Lesion site (*n*)	
Primary	3
Liver	70
Lung	12
Peritoneum	9
Other	7
RECIST classification (*n*)	
Partial response	27
Stable disease	55
Progressive disease	19
Age (years)	
Median	65
Range	17–83
Sex (*n*)	
Male	23 (58 %)
Female	17 (42 %)
Line of treatment (*n*)	
First	29 (72 %)
Second	11 (28 %)
Treatment regimen (*n*)	
FOLFOX	20 (50 %)
FOLFIRI	13 (33 %)
FOLFOX + bevacizumab	1 (3 %)
FOLFIRI + bevacizumab	4 (10 %)
FOLFIRI + panitumumab	1 (3 %)
Capecitabine	1 (3 %)

### Computed tomography

Each patient underwent a helical diagnostic CT scan with or without intravenous injection of contrast agent (depending on the lesion) 9 days on average (range 0–26 days) before the first FDG PET/CT scan, and after 6 to 8 weeks on therapy or sooner in patients with clinical suspicion of progression (three patients). Axial slice thickness was 3 or 5 mm depending on the CT scanner. The target lesions (no more than five per patient) were identified by a senior radiologist in a joint reading session with a nuclear medicine physician. Each lesion was analysed individually.

CT data were interpreted according to the RECIST 1.0 criteria [26] with the following restriction: only lesions clearly identified on both the baseline PET and diagnostic CT scans and with a diameter of at least 15 mm on the baseline diagnostic CT scan were analysed. Based on RECIST 1.0, lesions were classified as complete response to the treatment (CR), partial response (PR), stable disease (SD) and progressive disease (PD). Confirmation of SD status was obtained by an additional CT scan after a further 6 to 8 weeks.

### FDG PET/CT

Each patient underwent a baseline FDG PET/CT scan just before the start of chemotherapy and a second scan on day 14 after chemotherapy initiation. Patient preparation, imaging and reconstruction protocols were identical for serial scans. All FDG PET/CT images were acquired using a GE Discovery LS system, 60 min after injection of 4 MBq/kg. PET images were reconstructed with the built-in GE Healthcare Advance software, using the ordered subset expectation maximization algorithm [27] with two iterations and 28 subsets, and postfiltered with a 5.45-mm full-width at half-maximum (FWHM) gaussian function. The images were corrected for attenuation using the CT data and for scatter using a convolution-subtraction method [28]. CT was performed with a four-slice helical scanner (LightSpeed; GE Medical Systems). The tension was 120 kV and the current was determined by the Auto-mA GE algorithm and ranged from 30 mA to 200 mA. The other CT acquisition parameters were 0.5 s per CT rotation, with a pitch of 1.5 and a table speed of 15 mm per rotation. The matrix of CT images was 512 × 512 (0.98 × 0.98 mm pixel size) with a 5-mm slice thickness, and the PET matrix was 128 × 128 pixels of 3.91 × 3.91 mm with a slice thickness of 4.25 mm. Finally, the PET images were expressed in SUV, calculated using the expression:

1 $$ SUV\left( {\frac{g}{mL }} \right)=\frac{{Decay\,corrected\,uptake\,per\,volume\,unit\left( {\frac{{M{B_q}}}{mL }} \right)}}{{\frac{{Injected\,dose\left( {MBq} \right)}}{{Body\,weight(g)}}}} $$

### Characterization of tumour metabolism

Eight indices were used to quantify the tumour PET signal. All indices were calculated inside a large and manually defined volume of interest (VOI) centred on the lesion and including at least 50 % background activity. When required, the volume was adjusted so that only one hot region was contained in each VOI. These delineations were all performed by the same investigator using a research version of OWS software (Dosisoft, version 1.0.0.2.8).

#### Metabolically active volume

The lesion MV was obtained using the delineation method proposed by Nestle et al. [29]. The threshold value, *T*_bgd_, used for the delineation process was defined by:

2 $$ {{\mathrm{T}}_{\mathrm{bgd}}}=\alpha *\mathrm{SU}{{\mathrm{V}}_{70 }}+\mathrm{SU}{{\mathrm{V}}_{\mathrm{bgd}}} $$

To ensure connectivity between the voxels that define the MV, the largest region of connected voxels obtained after the application of this threshold was selected as the MV. The volume obtained using this algorithm depends on the mean uptake SUV_70_ in a region containing voxels with a value greater than 70 % of the maximum value in the VOI and on the surrounding background activity, SUV_bgd_. SUV_bgd_ was defined as the mean uptake in a 3-D shell region of 8 mm thickness placed at 16 mm from a region including all the contiguous voxels with uptake greater than 40 % of the maximum. To avoid the inclusion of irrelevant voxels, the boundaries of the background region were kept inside the VOI previously defined.

The *α* parameter in Eq. 2 was optimized using three acquisitions in a Jaszczak phantom composed of six spheres (volumes of 0.5, 1, 2, 4, 8 and 16 mL). The three acquisitions were performed using the same PET/CT scanner and acquisition protocol as for the patients. The only parameter varying between the three scans was the activity ratio between the sphere and the background regions. These ratios were 2.96:1, 5.88:1 and 10:1. A value of *α* = 0.3 was obtained by minimizing the average absolute error between the true sphere volumes and the volumes measured using *T*_bgd_ where the average error was calculated over all spheres and contrasts. We checked that this *α* value was robust with respect to the size of the spheres included in the optimization (results not shown).

#### SUV

Six SUV indices were calculated, including four indices without PVE correction and two with PVE correction.

- SUV_max_ was calculated as the maximum SUV in the tumour volume MV defined above.
- SUV_peak_ was computed as the average in a region of 3 × 3 × 3 voxels (1.75 mL) centred on the voxel corresponding to SUV_max._. Note that this is not identical to the SUL_peak_ index recommended in PERCIST.
- SUV_70_ was equal to the mean uptake in a region containing voxels with a value greater than 70 % of the maximum value in the large tumour VOI.
- SUV_mean_ was defined as the average SUV in the MV defined above.
- SUV_rc_ was equal to SUV_mean_ corrected for PVE using a recovery coefficient (RC) [23, 24]. The RC was calculated by convolving a binary mask corresponding to the MV with a 3-D gaussian function of FWHM equal to 7 mm. This 7-mm value was estimated by minimizing the mean square error in MV of the 18 spheres from the Jaszczak phantom images (six spheres × three contrast values). Spill-in was taken into account using SUV_bgd_ defined above.
- SUV_decon_ was obtained by performing a 3-D PET image deconvolution based on the Van Cittert iterative algorithm [22], using 12 iterations and a convergence rate set to 1. A mean SUV was then calculated in a 3-D region obtained using the region used to calculate SUV_70_ in Eq. 2.

#### Total lesion glycolysis

The TLG of each lesion was calculated as the product of MV with SUV_mean_ [8].

For each index and each patient, the percent change between the two scans was calculated for each lesion. For instance, with SUV_mean_, the percent change was given by:

3 $$ \varDelta SU{V_{mean }}\left( \% \right)=100\times \frac{{SU{V_{mean }}\left( {D14} \right)-SU{V_{mean }}\left( {Baseline} \right)}}{{SU{V_{mean }}\left( {Baseline} \right)}} $$

where D14 denotes the measurements performed on the PET/CT scan acquired after 14 days of treatment.

### Data analysis

The mean, standard deviation and range values over all lesions were calculated for each of the eight indices, at baseline and after 2 weeks of treatment. The agreement between indices in the baseline scans was evaluated using Bland-Altman plots [30]. The mean percent changes of the metabolic indices between baseline and day 14 were calculated for two groups of lesions:

1. The responding tumour group was defined as all lesions classified as PR or CR in the sense of the RECIST 1.0 criteria.
2. The nonresponding tumour group was defined as all lesions classified as PD or SD by RECIST 1.0.

Given that the statistical distributions of our indices in these two lesion groups departed significantly from normal distributions (Smirnov-Kolmogorov test), the significance of the differences between the medians of the percent change for the responding and nonresponding tumours was tested using a Wilcoxon signed ranks test with a significance level of 0.05.

To compare the performance of the eight indices in predicting the response to chemotherapy as later determined by RECIST, a nonparametric receiver operating characteristic (ROC) analysis was performed [31] using the responding and nonresponding tumour groups defined above. ROC curves were characterized by the area under the curve (AUC) and the significance of the difference between AUCs was tested using a nonparametric Friedman two-way analysis of variance by ranks [32].

Finally, the variability in each index was characterized using the coefficient of variation (CV, Eq. 4) of the absolute change between the two scans (Eq. 5).

4 $$ C{V_{index }}\left( {SD} \right)=\frac{{\sqrt{{\frac{1}{55}\sum {_{lesion=1}^{55 }} }}{{{\left[ {\varDelta inde{x_{lesion }}-\overline{{\varDelta index}}} \right]}}^2}}}{{\overline{{\varDelta index}}}} $$

with ∆index_lesion_ and $\overline{{\varDelta index}}$ defined as:

5 $$ \varDelta inde{x_{lesion }}=\left| {inde{x_{lesion }}\left( {D14} \right)-inde{x_{lesion }}\left( {Baseline} \right)} \right| $$

6 $$ \overline{{\varDelta index}}=\frac{1}{55}\sum {_{lesion=1}^{55 }} \left| {inde{x_{lesion }}\left( {D14} \right)-inde{x_{lesion }}\left( {Baseline} \right)} \right| $$

CV was calculated only for the 55 tumours classified as SD according to the RECIST 1.0 criteria. Indeed, for these lesions, the index change between the two scans would be expected to be negligible, and CV therefore represents mostly the variability of the index under similar conditions.

## Results

### Tumours

In the 40 patients, the mean number of lesions per patient was three (range one to eight). A total of 101 lesions selected according to the procedure outlined in the section Computed tomography were analysed (3 were primary lesions, 70 were located in the liver, 12 in the lungs, 9 in the peritoneum, and 7 at other various locations; Table 1). In these lesions, RECIST 1.0 classification yielded 27 PR, 55 SD and 19 PD lesions, and no CR lesions.

### Tumour metabolic volumes

**Fig. 1:**
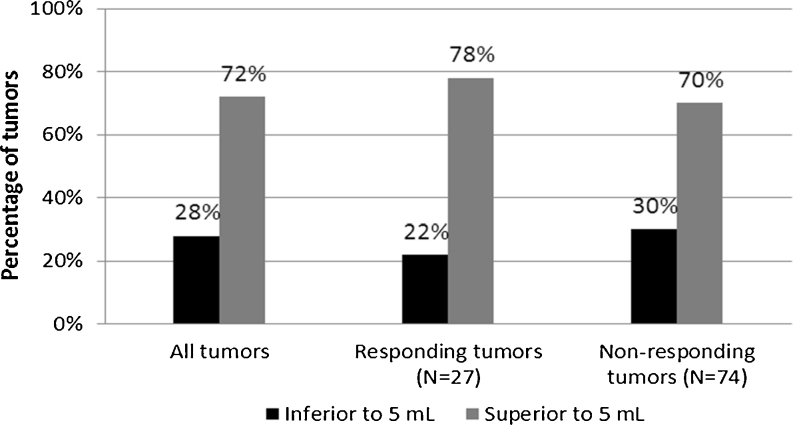
Percentages of tumours with a volume less than 5 mL and greater than 5 mL considering all tumours (*left*), only responding tumours (*centre*), and only nonresponding tumours (*right*)

The tumour MVs of the 101 lesions at baseline ranged from 1.0 to 382 mL (mean 34.4 ± 66.4 mL, median 8.5 mL). Figure 1 shows the distribution of tumour volumes at baseline for all tumours together, and also for the responding and nonresponding tumours separately. A chi-squared test showed that the distribution between the two volume groups at baseline (volume less than and more than 5 mL) was not significantly different between the responding and nonresponding tumours.

### Metabolic indices

The calculated values of the metabolic indices are given in Table 2. All indices showed a significant decrease after 2 weeks of chemotherapy (*p* < 0.05, two-sided Wilcoxon signed ranks test). The median percent changes in the indices between responding and nonresponding tumours were significantly different for all indices except MV (Mann-Whitney test).

**Table 2:** Calculated values of the eight indices for all lesions at baseline and on day 14 of treatment presented as means ± SD (min; max). The median percent changes after 2 weeks of treatment for responding and nonresponding tumours are also shown

Index	Baseline	Day 14	Change (%)	
			Responding tumours	Nonresponding tumours
SUV_mean_ (g/mL)	6.1 ± 2.2 (1.7; 12.4)	5.1 ± 1.9 (1.2; 10.4)*	−30.5 ± 15.2 (−72.4; −8.4)	−6.7 ± 29.9* (−56.5; 171.1)
SUV_peak_ (g/mL)	7.4 ± 3.4 (1.4; 21.3)	6.0 ± 2.9 (1.1; 18.0)*	−33.5 ± 17.0 (−74.0; −3.4)	−8.8 ± 37.1* (−8.8; 250.4)
SUV_70_ (g/mL)	8.3 ± 3.4 (2.1; 18.5)	6.9 ± 3.1 (1.3; 17.1)*	−31.0 ± 18.3 (−79.4; −1.5)	−7.3 ± 41.4* (−66.7; 276.3)
SUV_max_ (g/mL)	10.5 ± 4.3 (2.5; 22.3)	8.7 ± 3.9 (1.5; 21.1)*	−32.3 ± 18.3 (−78.1; 3.5)	−6.9 ± 37.3* (−66.7; 226.7)
SUV_rc_ (g/mL)	7.9 ± 2.7 (2.5; 14.6)	6.7 ± 2.5 (1.7; 12.9)*	−29.3 ± 18.2 (−76.6; −0.2)	−6.3 ± 30.0* (−56.3; 151.2)
TLG (g)	252.4 ± 559.3 (3.6; 3,644.9)	161.1 ± 293.6 (2.2; 1,574.3)*	−37.2 ± 40.7 (−81.2; 133.1)	3.3 ± 109.9* (−76.6; 834.1)
SUV_decon_ (g/mL)	11.7 ± 4.7 (3.1; 27.2)	9.8 ± 4.5 (1.8; 21.9)*	−28.0 ± 22.4 (−83.8; 6.7)	−6.6 ± 45.0* (−73.6; 261.6)
MV (mL)	34.4 ± 66.4 (1.0; 381.5)	27.4 ± 45.7 (1.0; 262.4)*	−4.9 ± 61.4 (−77.1; 218.2)	6.0 ± 59.2 (−74.9; 254.9)

*(column 3)*p* < 0.05, day 14 vs. baseline; *(column 5)*p* < 0.05, responding vs. nonresponding tumours.

### Bland-Altman plots

**Fig. 2:**
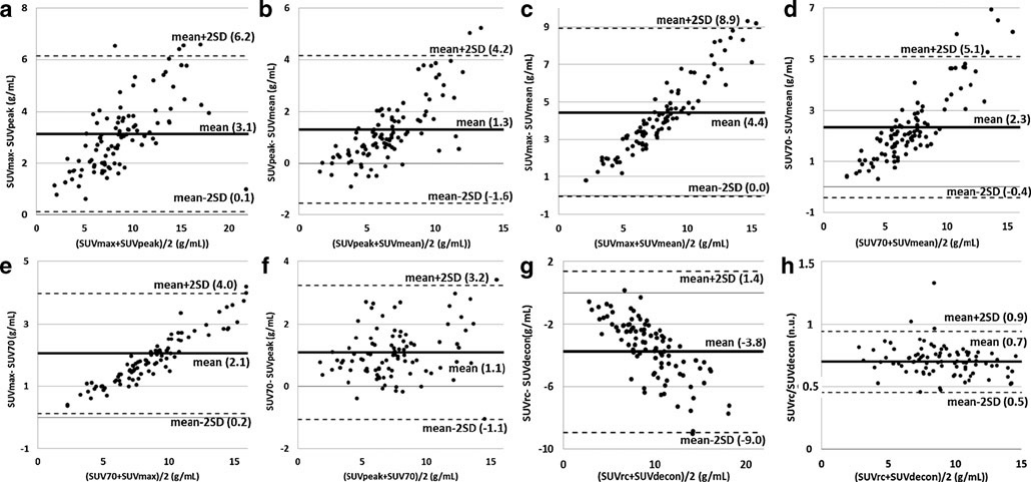
**a**–**g** Bland-Altman plots comparing indices for the 101 lesions before the first cycle of chemotherapy. The mean difference between each pair of indices and the mean ± 2SD are shown as solid and dotted lines, respectively, with the corresponding values in parentheses. **h** Plot of the ratio between SUV_rc_ and SUV_decon_ as a function of the mean, demonstrating that the two values are highly correlated

Figure 2 shows the Bland-Altman plots comparing the metabolic indices uncorrected for PVE (Fig. 2a–f) and comparing the two indices corrected for PVE (Fig. 2g). The strong linear relationship seen in most plots (except Fig. 2f) suggests that the two compared values were highly correlated. For instance, SUV_rc_ was, on average, 70 % of SUV_decon_ (Fig. 2h).

### ROC curve analysis

**Fig. 3:**
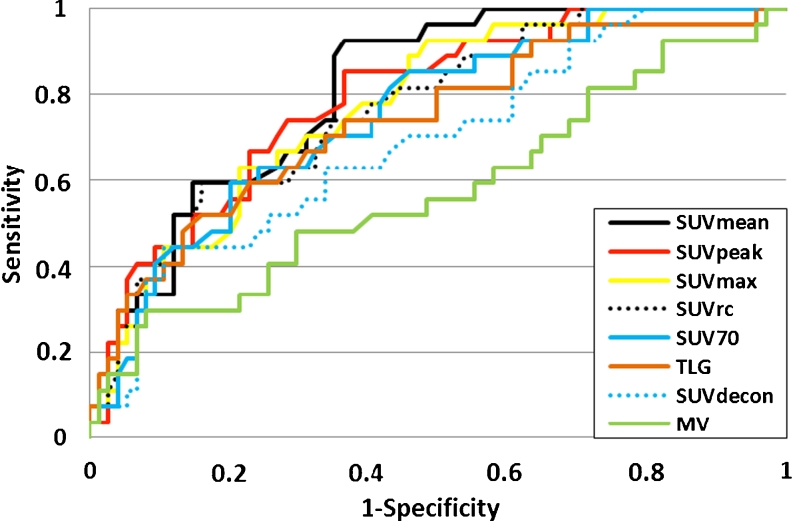
ROC curves for the SUV_mean_, SUV_peak_, SUV_max_, SUV_70_, SUV_rc_, SUV_decon_ and MV indices calculated from PET/CT scans performed on day 14 of therapy for the identification of responding (*n* = 27) and nonresponding (*n* = 74) lesions as defined by the RECIST 1.0 classification 6 to 8 weeks after treatment

Figure 3 shows the ROC curves for detecting lesion response to therapy for the eight indices. The AUCs (means ± SD) associated with the ROC curves were 0.81 ± 0.04 for SUV_mean_, 0.79 ± 0.05 for SUV_peak_, 0.77 ± 0.05 for SUV_max_, 0.77 ± 0.06 for SUV_rc_, 0.75 ± 0.05 for SUV_70_, 0.74 ± 0.06 for TLG, 0.69 ± 0.06 for SUV_decon_, and 0.58 ± 0.07 for MV.

A nonparametric Friedman two-way analysis of variance by ranks showed that the eight AUCs were not all identical. Comparisons of all pairs of AUCs using a multiple comparison procedure showed that only SUV_mean_, SUV_70_ and SUV_rc_ yielded an AUC significantly greater than that of MV (*p* < 0.05), while SUV_max,_ SUV_decon_, SUV_peak_ and TLG did not. SUV_decon_ showed poor classification performance, with an AUC substantially smaller than all AUCs associated with the other SUV indices. No other pairs of indices had significantly different AUCs.

### Coefficients of variation

The CVs for the change between the two scans for the 55 SD tumours were 0.72 for SUV_mean_, 0.82 for SUV_peak_, 0.76 for SUV_max_ and SUV_rc_, 0.75 for SUV_70_, 1.00 for SUV_decon_, 1.90 for TLG and 1.70 for MV.

## Discussion

The aim of this study was to clarify the impact of the index used for characterizing the metabolic activity of a lesion on FDG PET images when assessing the change in a lesion between a baseline scan and an early follow-up PET scan, performed 2 weeks after starting chemotherapy. In particular, the relevance of indices corrected for PVE in that context was investigated.

### Index values

All SUV-based indices are assumed to characterize the metabolic activity of a lesion. Yet the Bland-Altman plots (Fig. 2) demonstrate that one SUV index cannot be replaced by another. By definition, SUV_max_ is greater than SUV_mean_ and SUV_peak_, and Fig. 2a and c shows that the larger the SUV, the greater the difference between the two indices. Also, SUV_peak_ exceeded SUV_mean_ on average, although it could be smaller for small tumours, in which SUV_max_ sometimes corresponds to a voxel near the edge of the lesion and surrounded by low activity values “outside” the tumour. By definition, these low activity values are included when calculating SUV_peak_ but not when calculating SUV_mean_, therefor making SUV_peak_ lower than SUV_mean_. Figure 2 shows that for all SUV indices not corrected for PVE, the value depends more on the calculation approach (SUV_peak_, SUV_max_, SUV_70_ or SUV_mean_) for lesions with large SUV than for lesions with small SUV. The strong linear relationships seen on most Bland-Altman plots suggest that on average, one index can be roughly deduced from another using scaling factors, as illustrated in Fig. 2h. For instance, on average, SUV_max_ was 46 % greater than SUV_peak_, 71 % greater than SUV_mean_, and 24 % greater than SUV_70_.

Regarding the variability in the indices, the coefficients of variation suggest that all SUV indices not corrected for PVE had similar variability, which was between 2.1 and 2.4 times less than that of MV. The variability in TLG was the largest among all indices.

### PVE corrections

It is well known that PVE results in the largest underestimation of uptake in small tumours [19], especially in those whose dimensions are less than three times the spatial resolution in the reconstructed images. The spatial resolution in our PET images was about 7 mm FWHM, and hence tumours less than about 5 mL were strongly affected by PVE. This corresponded to 28 % of the 101 lesions. We could not restrict our analysis to these lesions because the number of tumours was then too low to demonstrate any significant difference between the responding/nonresponding tumour groups by the different indices. We thus considered all lesions in our analysis, checking that the proportions of small (≤5 mL) and large (>5 mL) tumours were not significantly different in the responding and nonresponding tumour groups (Fig. 1).

Two postreconstruction PVE corrections were tested. In the one using the Van Cittert iterative algorithm [22], the number of iterations should be carefully set to avoid a high increase in noise in the resulting images [33, 34]. We checked that our results remained unchanged in terms of statistical difference between differences in AUC when using 4 iterations instead of 12 in the Van Cittert algorithm. Another parameter of this PVE correction is the threshold (expressed as the percent of the maximum value in the tumour) used to calculate the PVE-corrected uptake in the deconvolved image. The 80 % threshold proposed by Teo et al. [22] was too high for our data and did not yield a VOI with spatially connex voxels. We used a 70 % threshold instead, i.e. exactly the same region as the one used to calculate SUV_70_ involved in the MV calculation so that SUV_70_ and SUV_decon_ only differed in terms of PVE correction. We also implemented the Lucy-Richardson [35] deconvolution and did not find any significant difference compared to the Van Cittert deconvolution, in agreement with Hoetjes et al. [17]. The other PVE correction we tested used RC and required an estimate of the spatial resolution in the reconstructed images. Our conclusions remained unchanged when assuming that the spatial resolution was 6 mm FWHM or 8 mm FWHM instead of the 7 mm value used in the results presented.

Because of PVE, the measured FDG uptake is strongly correlated with the tumour volume [19]. To assess the effectiveness of our two PVE corrections, we calculated the Pearson correlation coefficient between MV and each SUV index, at baseline and after one cycle of chemotherapy. This correlation coefficient was found to be much lower for the two PVE-corrected SUV indices (0.14 for SUV_rc_ and 0.09 for SUV_decon_) than for the uncorrected SUV (0.29 for SUV_mean_, 0.40 for SUV_peak_, 0.36 for SUV_max_ and 0.23 for SUV_70_), suggesting that the PVE corrections were effective. Unlike SUV_rc_, SUV_decon_ was not significantly linearly correlated with MV (*p* = 0.21). Looking closely at the differences between SUV_rc_ and SUV_decon_, SUV_decon_ was on average larger than SUV_rc_ (Table 2; Fig. 2g, h), which is also consistent with the fact that SUV_decon_ appeared to be more effective than SUV_rc_. The activity recovery produced by PVE correction in the tumour volume used to calculate SUV_70_, given by SUV_decon_/SUV_70_, was 1.43 (SD 0.22) when averaging over all lesions. The mean activity recovery produced by PV correction using the RC, given by SUV_rc_/SUV_mean_, was 1.33 (SD 0.14). These two close values confirm that the two PVE corrections were effective, and that the differences in results were mostly due to the regions in which the tumour activity was measured.

### Percent change in the indices between the two scans

The only metabolic index for which the mean percent change between the two scans was not significantly different between responding and nonresponding tumours was MV (Table 2). This is in agreement with the findings of Cheebsumon et al. [11] who found larger test–retest variability for MV than for SUV. This is partly because tumour delineation in PET is extremely challenging due to the low spatial resolution of PET compared to CT and to the relatively high noise level in PET images [36]. In addition, the chemotherapy-induced shrinkage of tumour volume is a slow process, with a decrease in volume after 2 weeks (one cycle) in responding lesions that is not yet significant. This explains at least partially why PVE correction in this setting does not increase the value of serial FDG PET scans in predicting response.

The ROC curves (Fig. 3) show that all SUV indices had similar performance in distinguishing between responding and nonresponding lesions as later classified by the RECIST 1.0 criteria, except SUV_decon_ which yielded an ROC closer to the diagonal line of no discrimination than the other SUV indices. The ROC curves also show that the change in MV provides some information to distinguish between responding and nonresponding lesions (AUC >0.5, *p* < 10^−5^). Even though it is far less informative than the SUV-based indices, removing this piece of information embedded in indices not corrected for PVE might be detrimental, as observed when comparing the ROC curves associated with SUV_rc_ and SUV_decon_ with those associated with SUV not corrected for PVE (Fig. 3). In particular, SUV_decon_ corresponding to the seemingly most effective PVE correction had a poorer classification performance than the SUV indices not corrected for PVE, as shown by the location of the ROC curve. This poor classification performance might also be explained by the high variability in SUV_decon_, compared to the other indices not corrected for PVE (see CV). Yet the TLG index had a much greater CV than SUV_decon_ and still a substantially higher AUC (0.74). This suggests that the poor performance of SUV_decon_ cannot be fully explained by its high variability. Comparing SUV_rc_ and SUV_mean_ alone (ignoring all other indices), which are two indices calculated from exactly the same voxels but with and without PVE correction, it appears that the PVE correction actually significantly reduced the AUC describing the classification performance (*p* = 0.02). The same was true when comparing only the AUC of SUV_decon_ and that of SUV_70_ (*p* = 0.03). By removing the volume information implicitly contained in SUV_mean_ or SUV_70_ because of PVE, SUV_rc_ and SUV_decon_ conveyed less information regarding the tumour response than when the volume information was implicitly included.

If the early change in MV is relevant for assessing tumour response, so should be the change in TLG, as MV is included in TLG. We indeed observed that TLG had classification performance not significantly different from that of the SUV indices not corrected for PVE. TLG also had better classification performance than SUV_decon_ (which does not include any volume information) despite a greater CV. This result confirms that what made SUV_decon_ poor in this classification task is the lack of embedded volume information rather than the high variability. We also investigated whether a TLG index calculated as the product of MV and an SUV corrected for PVE could better distinguish responding from nonresponding tumours than when TLG is based on an SUV not corrected for PVE. With TLG defined as the product of SUV_decon_ and MV, the AUC was 0.70 ± 0.07, while with TLG defined as the product of SUV_rc_ and MV, the AUC was 0.75 ± 0.06. Neither of these two values was significantly different from the AUC obtained with the original TLG (0.74 ± 0.06), suggesting that these different TLG definitions do not help in distinguishing responding from nonresponding tumours.

As we observed that the MV change provided some useful information for assessing tumour response, we also studied the tumour classification in a 2-D plan with change in SUV corrected for PVE on the *x*-axis and change in MV on the *y*-axis (results not shown). The tumour classification was not improved by this 2-D analysis and including MV information through a single index not corrected for PVE appeared more robust than considering independently the change in MV and in SUV corrected for PVE.

### Limitations of the study

In this investigation, we used the tumour classification obtained using the RECIST 1.0 criteria calculated 6 to 8 weeks after treatment initiation as a reference to determine the relevance of the tumour classification based on an early PET scan performed 2 weeks after treatment initiation. The indices calculated from the early PET scans and yielding the highest AUC therefore corresponded to the indices that best predicted the response seen 4 to 6 weeks later using the CT scan. RECIST is a surrogate end-point. Even if PET would have been of better predictive value than a late RECIST measurement for predicting outcome, any difference between metabolic information and the reference used here would be interpreted as a false-positive or false-negative result, and hence yield an AUC less than 1. Additional investigations regarding the role of PVE correction in tumour response assessment by considering progression-free survival or overall survival as end-points are still needed. Also, we did not validate the accuracy of the measurements performed by the different indices in the early PET scans, but only their ability to predict the anatomical response later seen on the CT scan.

About 75 % of the 101 lesions in our sample were classified as nonresponding lesions by RECIST 1.0. This implies that our results probably overestimated the specificity, and hence the ROC curves tended to be biased towards the line of no discrimination. Yet the lack of balance between the number of responding and nonresponding lesions was taken into account during the statistical analysis, and did not bias the comparative assessment of the different indices.

This study focused on the early metabolic tumour response. The role of PVE correction when characterizing the tumour response at later stages of therapy, i.e. when the shrinkage in tumour volume is large in responding tumours, still needs to be determined.

Last, our results were obtained for a particular lesion type in patients suffering from mCRC. Whether our results hold for different types of lesions remains to be demonstrated. In addition, it would be worth determining how other types of information drawn from the lesions, such as textural information [37] that has been recently demonstrated to better predict tumour response than SUV in oesophageal cancer lesions [38], would compare with the indices included in our study in the context of early assessment of response to therapy.

### Conclusion

In 40 patients with mCRC with 101 lesions (28 % less than 5 mL), we found that SUV_max_, SUV_mean_, SUV_peak_, SUV_70_ and TLG calculated in early PET scans (2 weeks after starting therapy) and compared with the corresponding baseline values all accurately predicted the late response (at 6 to 8 weeks on therapy) determined using the RECIST criteria in CT scans. Characterizing the change in lesion metabolic activity using an SUV corrected for PVE did not improve the discrimination of responding and non-responding lesions, possibly due to the fact that PVE correction removes most information pertaining to the MV. Considering the change in MV only between the baseline and early PET scans yielded a poor prediction of the response to therapy later identified on the CT scan.
